# Assisting Vascular Surgery with Smartphone Augmented Reality

**DOI:** 10.7759/cureus.8020

**Published:** 2020-05-08

**Authors:** Omar Aly

**Affiliations:** 1 General Surgery, Queen Alexandra Hospital, Portsmouth, GBR

**Keywords:** peripheral arterial diseases, augmented reality, image-guided radiosurgery, three-dimensional (3d) printing

## Abstract

Background

Augmented reality is a technology that expands on image-guided surgery to allow intraoperative guidance and navigation. Augmented reality-assisted surgery (ARAS) has not been implemented in the vascular field yet. The wealth of sensors found on modern smartphones make them a promising platform for implementing vascular ARAS. However, current smartphone augmented reality platforms suffer from tracking instability, making them unsuitable for precise surgery. Novel algorithms need to be developed to tackle the stability and performance limitations of mobile phone augmented reality.

Aim

The primary aim was to develop an ARAS system utilizing low-cost smartphone hardware for vascular surgery. The second aim was to assess its performance by evaluating the stability of its tracking algorithms.

Methods

We designed an ARAS system utilizing standard optical tracking (SOT) and developed a novel tracking algorithm: hybrid gyroscopic and optical tracking (HGOT) for improved tracking stability. We evaluated the stability of both tracking algorithms using a phantom model and calculated tracking errors using root mean square error (RMSE).

Results

The novel augmented reality system displayed a three-dimensional (3D) guidance model fused with the patient’s anatomy on a smartphone in real-time. The rotational tracking RMSE was 3.12 degrees for SOT and 0.091 degrees for HGOT. Positional tracking RMSE was 3.3 mm for SOT compared to 0.03 mm for HGOT. Comparing the stability of both tracking techniques showed HGOT to be significantly superior to SOT (p = 0.004).

Conclusion

We have developed a novel augmented reality system for vascular procedures. The development of HGOT has significantly increased the stability of a low-cost handheld augmented reality solution.

## Introduction

Background

Traditionally, the role of radiological imaging was limited to pre-operative diagnosis. Image-guided surgery extends the role of preoperative imaging to plan complex surgical procedures [1]. Image-guided surgery has been adopted in the neurological field for almost two decades [2]. Similarly, patient-specific anatomical models are being utilized to improve the surgeon’s understanding of liver vasculature and its relation to tumors. Studies utilizing three-dimensional (3D) reconstructions have shown improvements in tumor localization and the confidence of the surgeon while operating [3-5].

Augmented reality is a technology that expands on image-guided surgery, allowing intraoperative guidance and navigation. This technique integrates imaging information with the real-world surgical field to give the clinician what is colloquially known as “x-ray vision” [6]. At the core of augmented reality, systems are registration and tracking algorithms that map the orientation and position of the 3D guidance data to the patient's anatomy [7]. The rendering process draws the correctly oriented 3D guidance image and the clinician then perceives the augmented surgical field through a variety of technologies which include projectors, head-mounted displays, microscopes, medical displays, or smartphones [2, 8-9]. Augmented reality-assisted surgery has been implemented in the fields of hepatobiliary, neurological, and maxillofacial surgery and has been shown to improve clinical outcomes in neurological resections in a case-controlled study [9-11].

Vascular surgery, similar to the previously mentioned specialties, utilizes cross-sectional imaging for preoperative planning. The hybridization of radiological and surgical techniques is gaining traction as a method to tackle difficult vascular disease [12]. Intraoperative decisions can preemptively be planned with the wealth of data interpreted from cross-sectional imaging. Furthermore, anatomical variants can be identified preoperatively and appropriate preemptive measures can be applied intraoperatively.

Currently, there are no similar methods that allow the fusion of preoperative imaging to the surgical field in vascular surgery. Referencing preoperative imaging is challenging to achieve ergonomically in the context of a sterile operating field. This may discourage the surgeon from fully utilizing preoperative planning data [13]. We propose that a handheld smartphone can be developed into an augmented reality system that can be integrated easily into the surgical field. To implement this proposal, a set of tools and novel algorithms would need to be developed to tackle the stability and performance limitations of mobile phone augmented reality [14].

In this study, our primary aim was to develop an augmented reality-based system utilizing low-cost smartphone hardware as an ARAS implementation for vascular surgery. The second aim was to assess its performance by evaluating the stability of its tracking algorithms.

## Materials and methods

**Figure 1:**
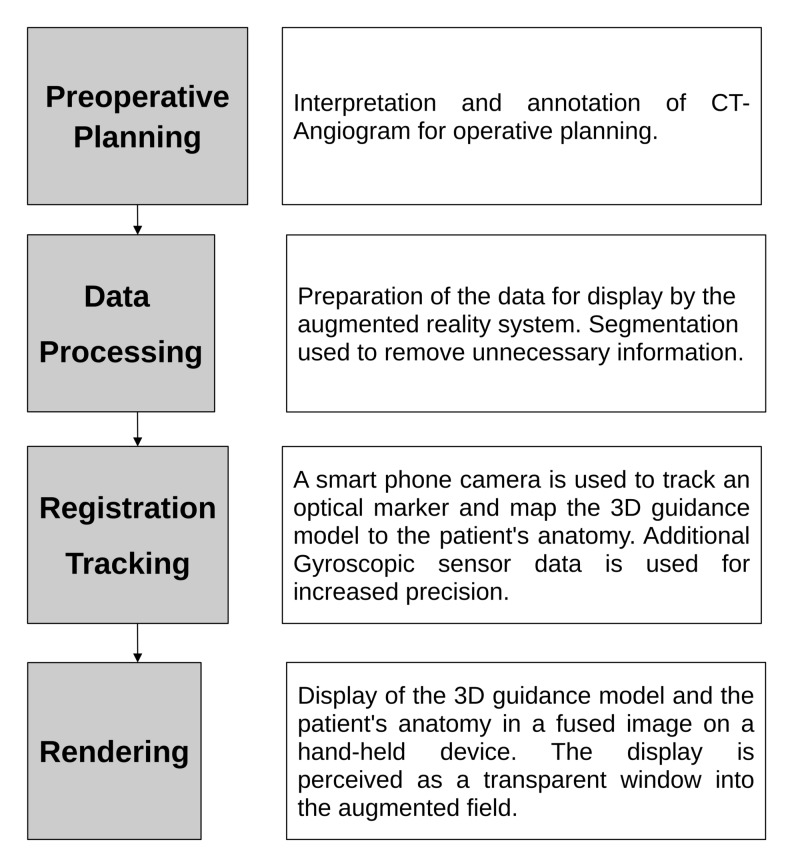
A flow chart illustrating the workflow implemented by the proposed ARAS system ARAS: augmented reality-assisted surgery; CT: computed tomography; 3D: three-dimensional

The initial phase of this study was developing a handheld augmented reality solution tailored for vascular ARAS. For this task, the smartphone platform was chosen as it is a low-cost method that can easily be integrated into any operating environment with the use of licensed sterile sleeves. The process of transforming preoperative cross-sectional imaging into a 3D guidance model that can be displayed on the smartphone augmented reality system is shown in Figure 1. Following the implementation of the augmented reality system, we evaluated the stability of two tracking algorithms to determine if it can provide a stable roadmap for ARAS.

Preoperative planning

**Figure 2:**
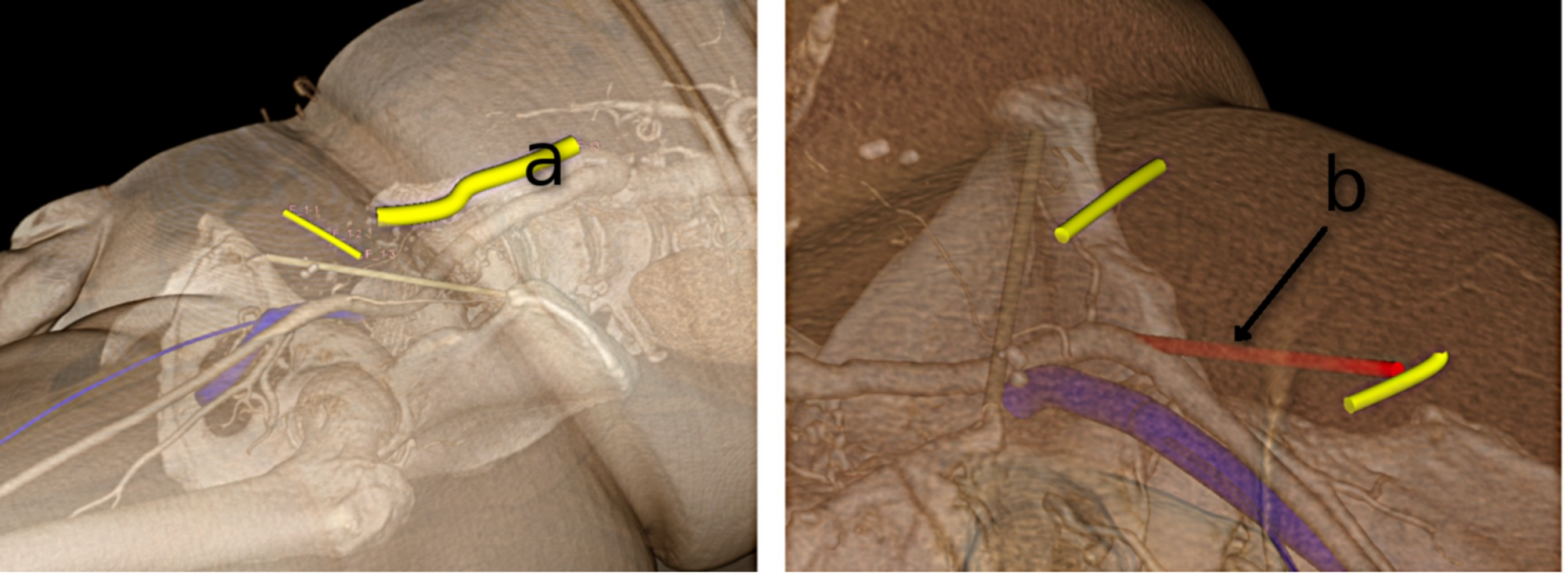
Volume rendered preoperative plans in 3D Slicer a) The first image shows the planned incisions for iliofemoral bypass; b) The second image shows the planned position of a secondary infra-inguinal incision for tunneling a sheath, enabling access for endovascular procedures in obese patients. The last image shows the annotation of the anatomical variation of the medial circumflex femoral artery.

The workflow for preoperative planning utilizes cross-sectional imaging in the form of computed tomography angiogram (CTA) Digital Imaging and Communications in Medicine (DICOM®) images (National Electrical Manufacturers Association, Rosslyn, VA). These images were loaded into the 3D Slicer (a free open-source software application for medical image computing) to allow examination and annotation of the patient’s anatomy [15]. Important landmarks and structures, such as the inguinal ligament and common femoral vein, were annotated using the 3D Slicer software. Tracking markers were placed on the skin to highlight optimal skin incisions that were tailored to the patient. Examples of preoperative plans in the 3D Slicer are shown in Figure 2.

Data processing

Following the planning phase, the volumetric data from the CTA is automatically segmented using an intensity threshold level to extract the patient's contrast-enhanced vasculature and bony anatomy. This segmented volume is converted to a 3D polygonal mesh using the marching cubes algorithm. We elected to use a polygonal rather than a volumetric guidance model as this can be rendered in real-time on the smartphone. Annotation markers placed by the clinician are also converted into polygonal models. The origin of the 3D guidance model is set to the location that the tracking marker will be placed on the patient. 

Registration and real-time tracking

**Figure 3:**
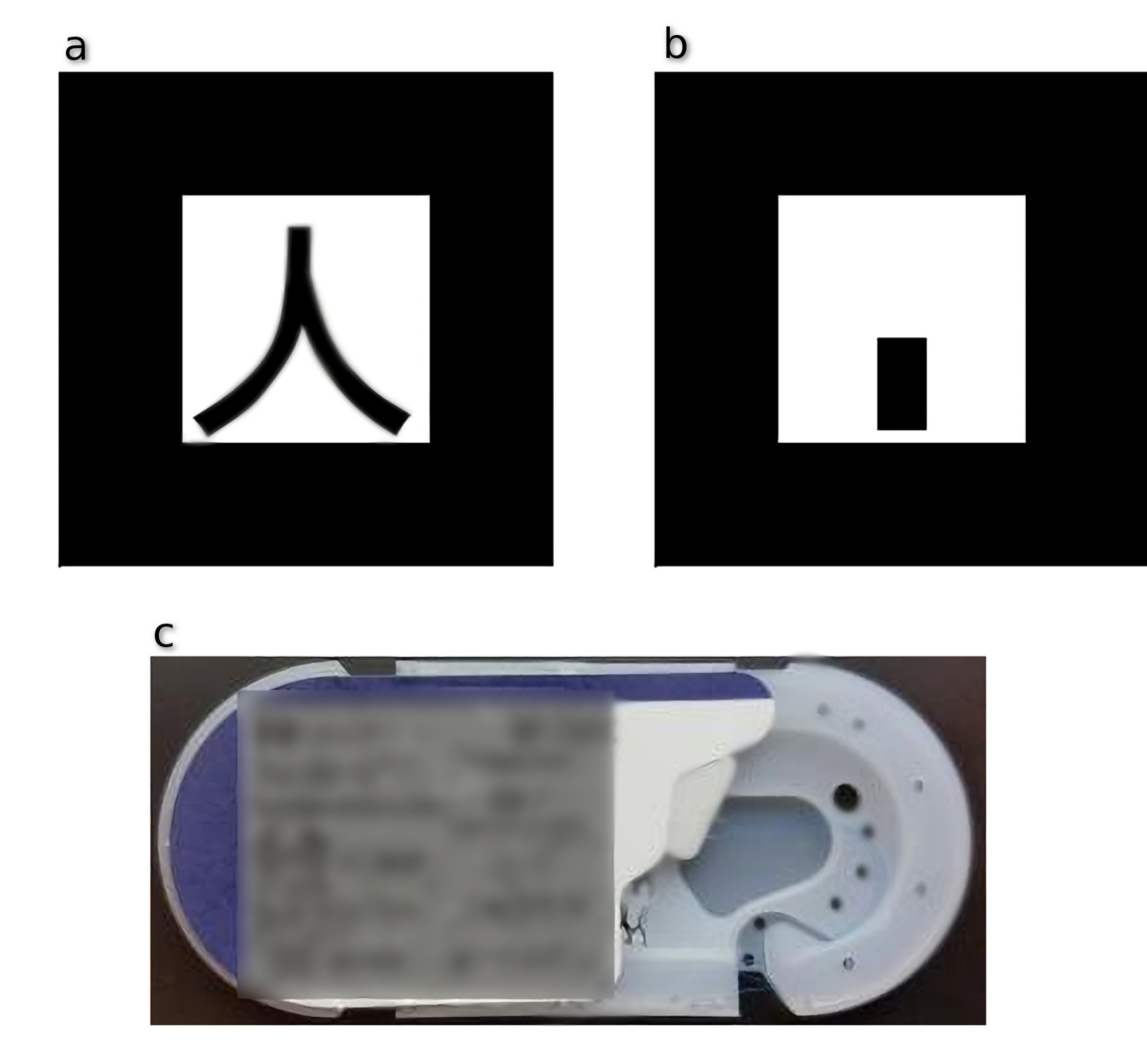
Examples of optical tracking markers The top images show traditional paper tracking markers (a, b). The lower image shows a sterile suture pack that is recognized as a tracking marker in this implementation (c).

At the core of the ARAS software are the tracking and registration algorithms, a process where 3D guidance models are positioned and oriented correctly in relation to the patient's anatomy. Since we were aiming to design a portable non-intrusive tracking system, we elected to use monoscopic optical tracking using the high-resolution camera found in smartphones. The tracking software was built upon the Vuforia Software Developer Kit (SDK) (PTC, Inc., Boston, MA, USA) for mobile devices [16]. Rather than utilizing traditional augmented reality tracking markers, we opted to use natural feature tracking to train the tracking software to identify objects commonly found in the sterile operating field (Figure 3). Suture packets provide an ample number of distinct optical features that are well-detected by the tracking algorithms. The tracking algorithm was run in high definition (1,920 x 1,080) at 30 frames per second. In this study, we evaluated two tracking methods, standard optical tracking (SOT) from the Vuforia SDK and our own hybrid gyroscopic and optical tracking (HGOT) method. 

Standard optical tracking (SOT) algorithm

The Vuforia planar marker tracking algorithm was used for the SOT algorithm. The pubis symphysis, a consistent bony prominence, was used as the location and origin for the tracking marker in the 3D coordinate system. The virtual X, Y, Z coordinates represent the patient’s coronal, sagittal, and transverse planes, respectively.

The registration process was initiated by the user focusing the camera on the pubis symphysis tracking marker. The inverse of the tracking marker's transformation matrix was applied to the virtual camera. This process couples the visual position and orientation of the 3D guidance model aligned to the patient. Real-time tracking continues to ensure that both the patient's anatomy and the 3D model remain in identical positional alignment and orientation. 

We elected to implement optical tracking using planar trackers as they have displayed excellent performance with a low magnitude of error if used within well-defined distances and viewing angles [17-18]. 

Hybrid gyroscopic and optical tracking (HGOT) algorithm

Throughout this project, several performance limitations of SOT became apparent. To overcome these limitations, a novel algorithm, HGOT, was developed to provide increased tracking stability. This utilized an optical marker tracking, together with the smartphone's high precision internal gyroscope.

The algorithm initially registers the patient's sagittal orientation, as it is assumed the operating table and patient's orientation will not change following the initial registration. The screen space position of the model is calculated based on the optical tracking marker's position only. Finally, the orientation of the virtual camera is tracked directly from the smartphone internal gyroscope, which adjusts the visible X and Z orientations of the 3D guidance model. The gyroscope is recalibrated periodically to counter the effects of gyroscopic drift using accelerometer data. 

Rendering of visualization

Following the calculation of the transformation of the 3D guidance model and virtual camera through the tracking algorithm, the 3D guidance model is rendered in real-time on the smartphone. The scene is composed by combining the smartphone camera output with the 3D rendering to allow the phone’s display to be perceived as a transparent window into the augmented reality operative field.

Initial testing

The feasibility of the augmented reality system in displaying the 3D guidance model onto human anatomy was evaluated qualitatively on a volunteer subject. The displayed positions of the virtual bony landmarks and vascular structures were compared to the subject's anatomy using palpation.

Evaluation of tracking system stability

We objectively evaluated the performance of both SOT and HGOT through a phantom model to identify the most suitable algorithm of our intended application. The phantom model consisted of a human torso model with an attached optical tracking marker at the pubis symphysis, a tripod to stabilize the smartphone tracking device at a defined distance and position from the tracking model, and a 3D guidance model loaded on the smartphone which matched the dimensions of the groin and torso model. To evaluate the performance of the handheld tracking algorithms in conditions similar to usage, we tested the smartphone tracking performance at incremental distance intervals starting from 5 cm up to 30 cm from the tracking marker. The tracking data was sampled over 5 seconds (n = 150 at 30 frames per second).

The generated tracking position and rotation values were transferred from the smartphone to a desktop computer for statistical analysis. The generated tracking position and rotation values were compared to predicted values derived from the position of the camera and phantom model in the testing apparatus. The root mean square error (RMSE) was calculated from the tracking data error and the statistical significance between SOT and HGOT stability was analyzed with the Mann-Whitney U test. An alpha threshold of less than 0.05 was considered to be statistically significant.

To evaluate positional tracking stability, we calculated the RMSE in the tracked position of a clinically significant area of interest away from the origin of the coordinate system. For this clinical application, we chose the bifurcation of the common femoral artery. 

## Results

**Figure 4:**
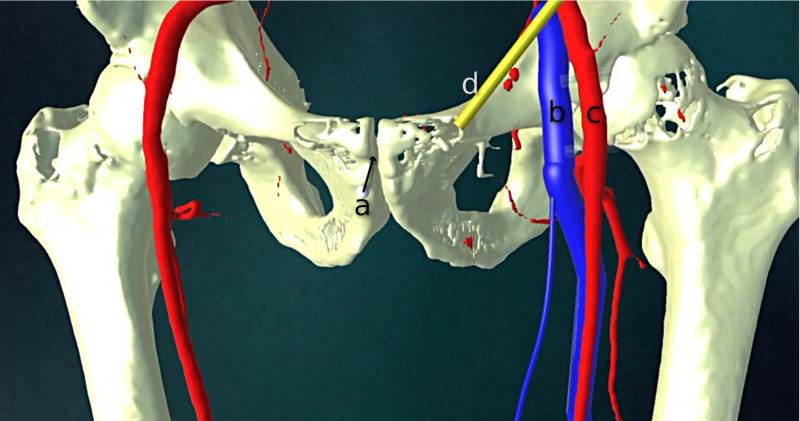
Raw rendering of three-dimensional (3D) guidance model prior to registration, tracking, and compositing a) the virtual position of the tracking marker; b) common femoral vein; c) common femoral artery; d) inguinal ligament

**Figure 5:**
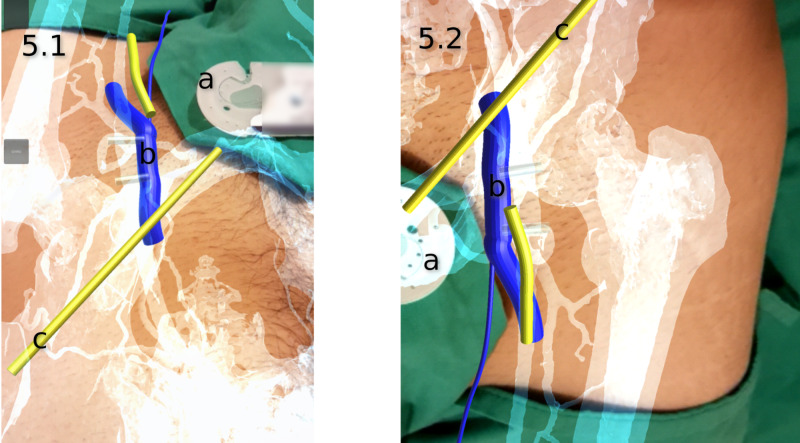
A screen capture from the smartphone showing the augmented reality viewport following registration, tracking, and compositing from multiple viewing angles The tracking algorithm tracked the marker in both the craniocaudal (Figure 5.1) and caudocranial (Figure 5.2) orientations. a) tracking marker; b) common femoral vein; c) inguinal ligament

**Figure 6:**
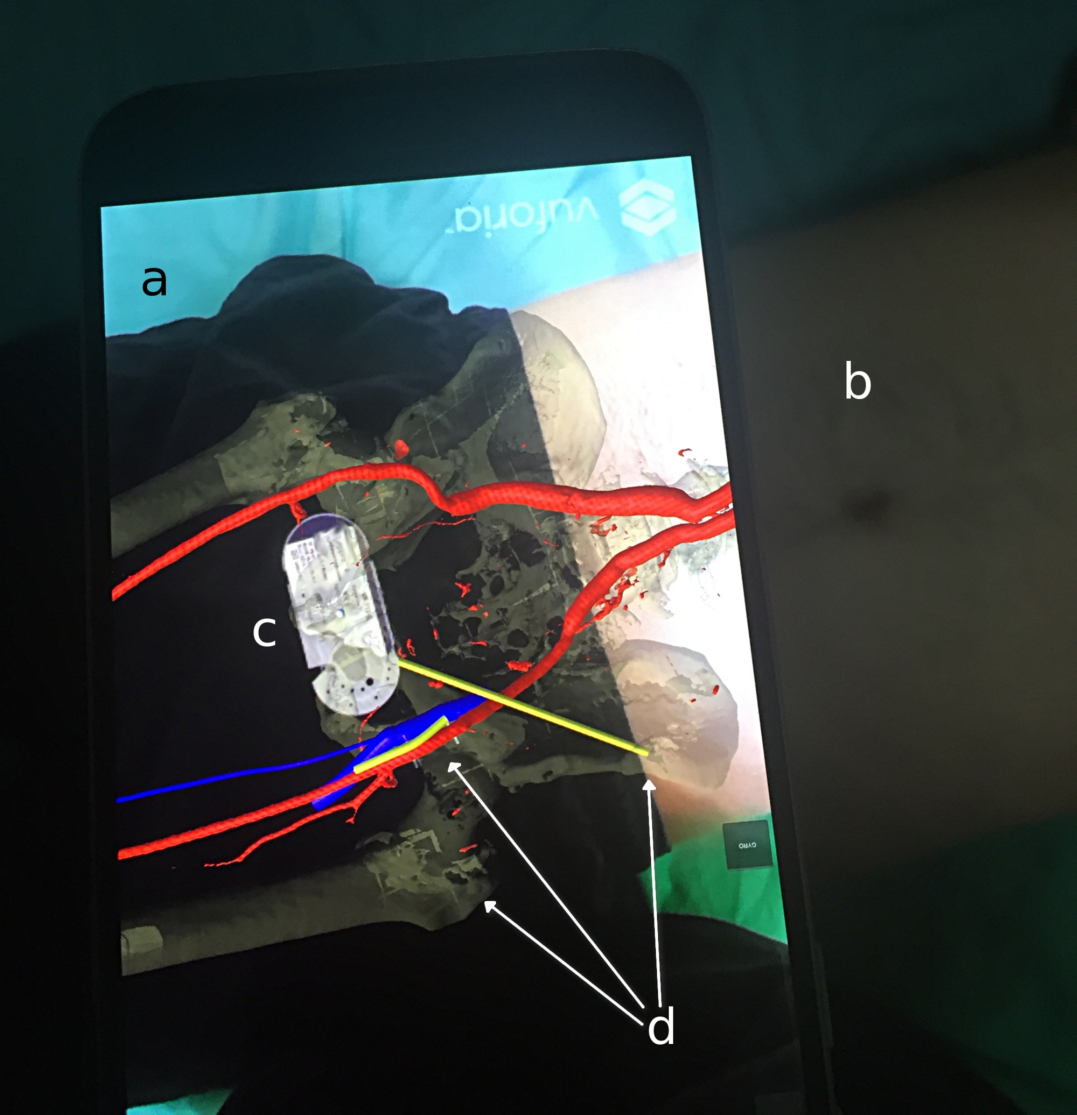
A photograph showing the augmented reality system from the perspective of the user; the 3D guidance model and composited image are rendered on the smartphone display in alignment with the subject's anatomy. a) augmented reality view on the smartphone; b) subject being tracked; c) tracking marker; d: key anatomical landmarks aligned with subject's anatomy

The methods and software described in the previous section were successfully able to display the 3D guidance model composed of the reconstructed vascular and bony anatomy fused with the real-time footage of a model patient's anatomy (Figures 4-6). 

Stability of rotational tracking

**Figure 7:**
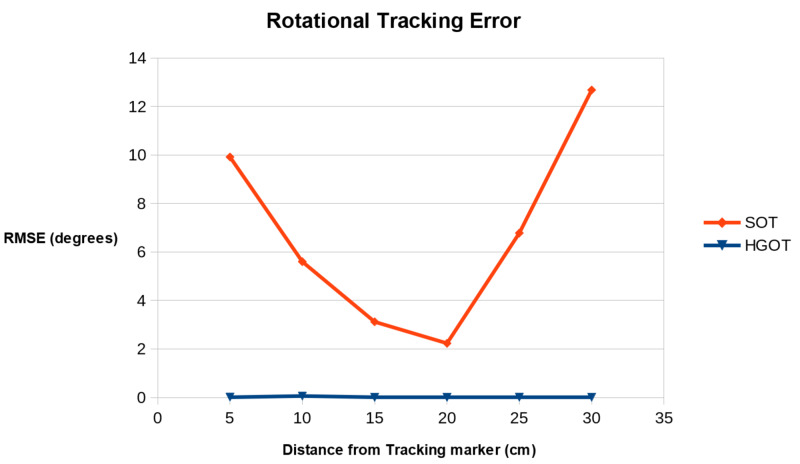
Comparison of rotational tracking error RMSE for SOT and HGOT tracking methods over increasing viewing distance intervals HGOT: hybrid gyroscopic and optical tracking; RMSE: root mean square error; SOT: standard optical tracking

The error in rotational tracking from both tracking systems was obtained as the RMSE in degrees and is shown in Table 1. Stability results at incremental distances from the tracking marker are shown in Figure 7. Both tracking systems performed optimally at 15 cm from the tracker with an RMS of 3.12 degrees and 0.091 degrees for SOT and HGOT, respectively. Despite the magnitude of rotational tracking error in SOT being low (2.23 degrees at 20 cm), this still resulted in a perceivable jitter in the position of the femoral bifurcation displayed by the 3D guidance model. Errors in HGOT tracking were of a magnitude too small to be perceived as jitter by the user in this application. Comparing performance across all distance ranges showed the stability of rotation tracking from the HGOT system to be significantly superior to SOT (p = 0.004).

**Table 1:** Rotational Tracking Stability Results at Distances Encountered in Handheld Tracking Hybrid gyroscopic and optical tracking (HGOT) was significantly more stable than standard optical tracking (SOT) (p = 0.004). RMSE: root mean square error

	Distance from Tracking Marker (cm)					
	5 cm	10 cm	15 cm	20 cm	25 cm	30 cm
SOT Rotational Tracking RMSE (degrees)	9.91	5.61	3.12	2.23	6.78	12.68
HGOT Rotational Tracking RMSE (degrees)	0.018	0.062	0.091	0.071	0.014	0.0083

Stability of positional tracking

**Figure 8:**
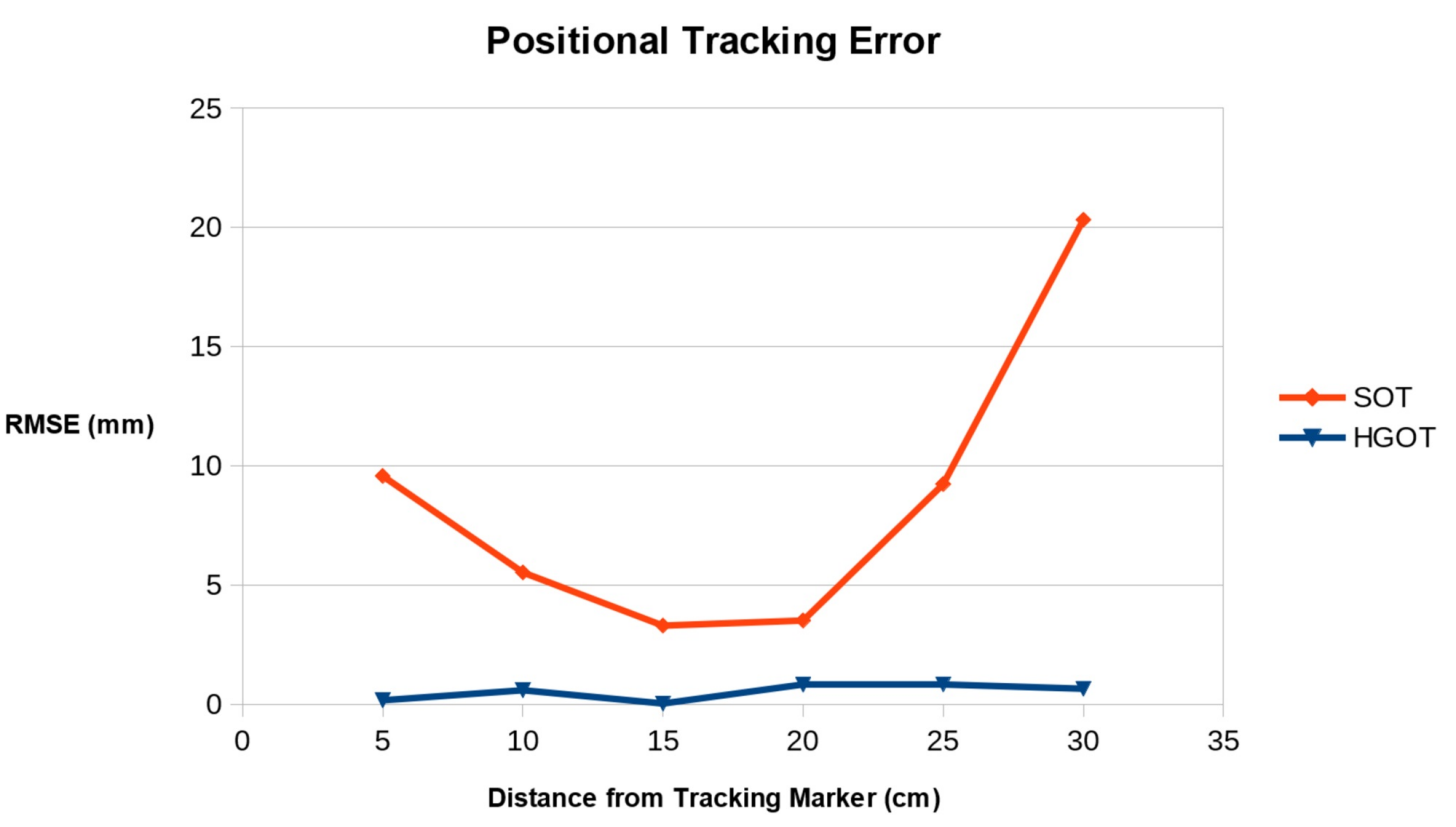
Comparison of rotational tracking error (RMSE) for SOT and HGOT tracking methods over increasing viewing distance intervals HGOT: hybrid gyroscopic and optical tracking; RMSE: root mean square error; SOT: standard optical tracking

The error in positional tracking data for the femoral bifurcation from both tracking systems was obtained as the RMSE in millimeters (mm) and is shown in Table 2. Stability results at incremental distances from the tracking marker are shown in Figure 8. SOT performed optimally at 15 cm with an RMS of 3.3 mm. However, this was still inferior to HGOT which had a root mean square (RMS) of 0.03 mm. At 30 cm from the tracking marker, SOT failed to provide suitable orientation data for augmented reality as the RMSE was 20.3 mm. Comparing performance across all distance ranges showed the stability of positional tracking from the HGOT system to be significantly superior to SOT (p = 0.004).

**Table 2:** Positional Tracking Stability Results at Distances Encountered in Handheld Tracking Hybrid gyroscopic and optical tracking (HGOT) was significantly more stable than standard optical tracking (SOT) (p = 0.004). RMSE: root mean square error

	Distance from Tracking Marker (cm)					
	5 cm	10 cm	15 cm	20 cm	25 cm	30 cm
SOT Positional Tracking RMSE (mm)	9.57	5.52	3.29	3.51	9.23	20.32
HGOT Positional Tracking RMSE (mm)	0.168	0.589	0.033	0.825	0.837	0.652

Previous augmented reality research has shown that SOT tracking performs optimally at low distances from the tracking marker (Tables 1-2) [17-18]. However, our results diverged from previous research. The tracking stability for SOT degraded at very low distances from the tracking marker (5 cm). This may be due to part of the tracking marker being outside of the camera's field of view at close ranges. The major limitation of SOT is that the optical tracking marker is usually placed away from the structure of interest in order not to obstruct the surgeon's view of the operative field. This distance from the target of interest significantly increases the rotational tracking error in traditional augmented reality algorithms. 

## Discussion

Intraoperative navigation and visualization is currently expensive and cumbersome to integrate into crowded operating rooms. Following the initial capital investment in software and hardware, the initial setup at the start of the procedure can incur indirect costs due to increased operative time. These time and monetary costs have discouraged surgeons from adopting new technologies with unestablished effects on clinical outcomes. Our solution aims to utilize the rendering and tracking capabilities of modern smartphones to provide a low-cost, non-intrusive solution to augmented reality-guided vascular surgery. We have shown that combining optical and gyroscopic tracking (HGOT) can provide precise tracking for augmented reality in a compact low-cost hardware solution. 

Stability of the augmented reality solution

Our HGOT method subdivides the tracking algorithm into its subcomponents (translation, rotation, and scale) and allocates them to the best-suited hardware sensor found on modern smartphones. Current smartphones with gyroscopes are able to achieve a rotational tracking resolution of 2,000 degrees per second. The addition of gyroscopic rotational tracking in the HGOT algorithm has greatly reduced the instability which plagues SOT at longer viewing distances. This allows a handheld device to remain versatile in a dynamic operating environment.

User experience

Currently, there are several methods of visualizing augmented reality data ranging from projectors, head-mounted displays, and monitors used for endoscopic procedures. From the surgeon's perspective, the optimum device should facilitate the procedure and cause minimal disruption to the operation. The advantages of constant real-time guidance should also be balanced against the detrimental effects on the surgeon’s comfort and vision inflicted by these devices [19]. 

We believe our method of a handheld device will be familiar to vascular surgeons as they currently utilize a handheld Doppler for intraoperative navigation and would avoid the disadvantages of other display methods. However, unlike the handheld Doppler device, our implementation has the added advantage of not being in contact with the operative field. This allows the surgeon to continue operating while the device is held by the assistant in the form of a transparent window into the augmented reality operative field.

Limitations

The main limitation of current registration and tracking systems is the ability to track mobile or deformable anatomy. Bony anatomy has been registered with a high degree of success in orthopedic and neurosurgical systems. On the other hand, intra-abdominal structures that move and deform with ventilation have proven to be a challenge. In our proposed application, registration of deformation is not a major issue as vascular anatomy is relatively fixed and the patient is not repositioned intraoperatively. However, the effects of soft tissue deformation due to intraoperative retraction may reduce the stability of the system. 

Clinical application

The routine use of image-guided surgical technology is debatable as the expert surgeon may find the technology to be unnecessary or intrusive to the flow of the operative procedure. In our proposed application, which focuses on inguinal surgery, the anatomy of the femoral triangle and its vessels is familiar territory to the vascular surgeon. However, possible anatomical variation, obesity, scarring from previous surgery, and radiotherapy can prove challenging, even to experienced surgeons. In these situations, augmented reality can provide patient-specific guidance as an adjunct to operative experience.

Clinical studies comparing the outcomes of navigated surgery to surgery without its support have lagged behind technological developments. This ultimately can discourage clinicians from adopting new technologies. In theory, technical accuracy and good planning are surgical principles that can be enhanced by augmented reality guidance; however, they do not guarantee improved clinical outcomes. Since the operative approach and accurate incision planning have been shown to influence the incidence of wound complications in vascular operations, it is possible that augmented reality-guided vascular surgery can improve clinical outcomes [20-22]. This will require validation through future clinical studies. 

Future work

A barrier to implementation of augmented reality tracking is the space requirement for the optical tracking markers; optical tracking markers need to be large enough to be detected by the tracking cameras while striving not to obstruct the operative field. One potential solution is the use of computer vision algorithms to identify hand-drawn markers on the patient's skin. Simultaneous Localization and Mapping (SLAM) is an emerging technology that can provide “marker-less” registration [23]. We believe SLAM registration, combined with gyroscopic tracking methods, could provide a robust and less intrusive solution for ARAS. 

## Conclusions

We have developed a novel augmented reality system that utilizes smartphone hardware for augmented reality-assisted vascular surgery. The use of both the camera and gyroscopic sensors found in smartphones has allowed the HGOT algorithm to significantly increase the tracking and registration performance of a low-cost, handheld, augmented reality solution.
